# Femtosecond laser-assisted synthesis of silver nanoparticles and reduced graphene oxide hybrid for optical limiting

**DOI:** 10.1098/rsos.171436

**Published:** 2018-07-04

**Authors:** Yang Yu, Lihe Yan, Mengmeng Yue, Huanhuan Xu

**Affiliations:** Key Laboratory for Physical Electronics and Devices of the Ministry of Education and Shaanxi Key Laboratory of Information Photonic Technique, School of Electronics and Information Engineering, Xi'an Jiaotong University, Xi'an 710049, People's Republic of China

**Keywords:** laser materials processing, carbon materials, nanomaterials, nonlinear optics

## Abstract

Reduced graphene oxide (rGO) functionalized with silver nanoparticles (Ag NPs) is prepared using a femtosecond laser ablation in liquids method. By ablating the mixed aqueous solutions of silver nitrate and graphene oxide (GO) using femtosecond laser pulses, Ag ions and GO are simultaneously reduced and well-dispersed Ag NPs supported on rGO are obtained. The effect of laser power, irritation time and Ag ion concentration on the optical property and morphology of the products are systematically studied. The nonlinear optical responses of the functionalized graphene are studied using a nanosecond Z-scan technique. The rGO hybrid shows an enhanced nonlinear absorption (NLA) effect compared with GO and rGO, and thus exhibits an excellent optical limiting (OL) property with very low activating threshold, which is estimated to be about 0.38 J cm^−2^. The enhanced NLA effect in rGO hybrids makes it possible to fabricate solid-state optical limiter, improving the practicality of graphene materials in the OL area.

## 1. Introduction

The intensive developments and widespread applications of laser techniques sets a problem of protecting detecting equipment and human eyes from powerful laser radiation [1,2]. Nonlinear optical (NLO) limiters are designed for this problem. In the past decades, significant research efforts have been directed towards developing efficient optical limiting (OL) materials in an attempt to realize the protection from laser beams, although their practical applications still present a great challenge. For example, carbon black suspensions (CBS) [3,4] and carbon nanotubes (CNTs) [5–7] can attenuate intense laser by a nonlinear scattering (NLS) effect in a broadband wavelength range. But the activating threshold of the OL behaviour is considered to be too high and the NLS mechanisms are effective against laser pulses shorter than a few nanoseconds owing to their required breakdown time.

Graphene has attracted significant research interest owing to its excellent electronic, optical and mechanical properties [8,9]. As the interband optical transitions in graphene are independent of incident light wavelength over a wide range, it has exhibited unique NLO properties [10,11]. Since it was first reported by Wang *et al*. [11], much work has been done to study the OL properties and mechanisms of graphene, graphene oxide (GO) and graphene nanocomposites [12,13]. As has been demonstrated in CBS and CNTs, the main OL mechanisms of graphene suspensions were attributed to the NLS effect, in which solvent microbubbles and/or microplasmas were formed at a high input light fluence, inducing the attenuation of the incident laser beams. However, as there was no solvent and breakdown of the material in solid-state samples, the NLS effect was ineffective, retarding its practical application in optical limiter design. To this end, graphene nanocomposites with strong nonlinear absorption (NLA) captured researchers' attention.

The abundant oxygen-containing groups in GO make it possible for chemical functionalization of the material [14,15]. Various multi-functional materials such as organic materials, dye molecules and dielectric have been attached to GO to enhance the NLO properties [16–20]. Among these materials, metal nanoparticles (NPs) modified reduced graphene oxide (rGO) attracts much attention [21,22]. Though many synthesis strategies based on chemical methods have been explored to fabricate different kinds of graphene hybrids, the involvement of environmentally hazardous chemicals and complex chemical processes largely restrict both the versatility and flexibility of the hybrid graphene materials [17,18,23]. Laser ablation in liquids (LAL) methods can provide a green one-step synthesis strategy of graphene nanocomposites [24–28]. Owing to its ultra-short pulse duration and ultra-high peak power [29–32], when femtosecond laser irradiates on GO aqueous solution containing metal ions, plasma plumes are produced and reactions between these species result in NPs formation, as well as the reduction and doping of GO. Using this method, Tan *et al*. [13] fabricated rGO hybrids decorated with silver (Ag) NPs with nanometre sizes. Although the NLO response of the hybrids for femtosecond laser has been reported before, the controllable synthesis of Ag NPs/rGO composites and their OL response for more widely used nanosecond laser pulses still needs further investigation.

In this paper, rGO functionalized with Ag NPs nanocomposites are synthesized using a femtosecond LAL method. The prepared Ag NPs/rGO composites are studied using absorption spectroscopy, transmission electron microscopy (TEM) images, Raman spectra and X-ray photoelectron spectroscopy (XPS). The effect of laser power, irritation time and Ag ion concentration on the optical property and morphology of the products are systematically studied. From the view point of practical applications, the OL properties and mechanisms of Ag NPs/rGO composites are studied using the nanosecond laser Z-scan technique, especially the NLA contribution to the OL behaviour of the material are investigated. The results indicate that Ag NPs/rGO exhibits better OL properties than rGO, which can be attributed to the enhanced NLA and NLS effect in the composites. The enhanced NLA effect in the composites might promote the practical application of graphene materials in the fabrication of solid-state optical limiters.

## 2. Material and methods

In our experiments, the LAL method is used to synthesize the Ag NPs/rGO hybrids. Two milligrams of GO were dispersed in 10 ml distilled water by sonicating for 1 h until a uniform yellow solution was obtained. Then, AgNO_3_ with different concentrations was added into the solution and sonicated again. During the reduction process, a Ti : sapphire femtosecond laser system with central wavelength of 800 nm, pulse duration of 150 fs and repetition rate of 1 kHz was used. The laser beam is focused into the suspension by a 100 mm lens. The laser power varied from 100 to 500 mW. During the laser reduction process, a magnetic stirrer is used to make the solution irradiated homogeneously.

To study the morphology of the products, TEM and high-resolution TEM (HRTEM) images were carried out using a JEM-ARM200F microscope. Raman spectra were measured using an HR-800 Laser Raman spectrometer with excitation wavelength at 632 nm. XPS were done via an AXIS ULtrabld XPS spectrometer. UV–Vis absorption spectra were obtained using a UV-2600 spectrophotometer.

**Figure 1.**
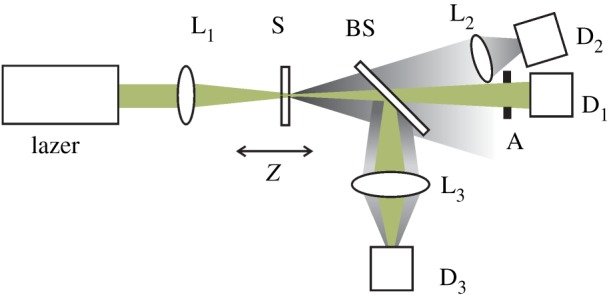
Z-scan experimental set-up. L, lens; S, sample; A, aperture; D, detector.

The OL properties of different graphene dispersions were studied using a Z-scan system as illustrated in figure 1. A Q-switched Nd^3+^ : YAG laser emitting 10 ns pulses with a pulse repetition rate of 10 Hz at 532 nm was used. All the dispersions are filled in 2 mm thick quartz cells. The laser pulses were focused into the sample using a lens (L_1_) with 20 cm focal length. The transmitted laser power as a function of the *z*-position was recorded by the detector (D_1_). To study the OL mechanisms, a fraction of the scattered light was collected using a convex lens (L_2_) at approximately 15° in the forward direction from the beam axis, and detected by a photodiode (D_2_). On the other hand, a part of the output beam from the sample including amounts of scattered light was collected by a lens and detected by the detector (D_3_). By measuring the incident power dependence of the nonlinear transmittance of the samples, the contributions of the NLA effect to OL behaviours can be confirmed.

## 3. Results and discussion

### 3.1. Synthesis and characterizations of silver nanoparticles/reduced graphene oxide composites

**Figure 2.**
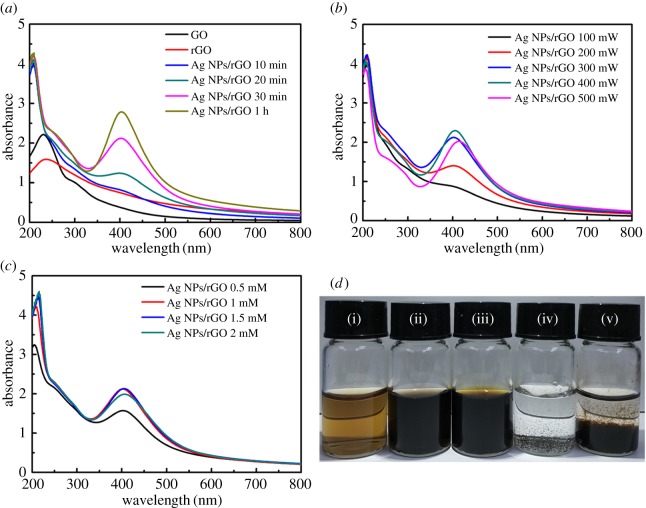
Absorption spectra of the products prepared (*a*) with different irradiation time (Ag ion concentration fixed at 1 mM, and laser power fixed at 300 mW), (*b*) with different laser power (irradiation time fixed at 30 min, and Ag ion concentration fixed at 1 mM) and (*c*) with different Ag ion concentrations (laser power fixed at 300 mW, irradiation time fixed at 30 min). (*d*) Photographs of (i) GO, (ii) rGO, and (iii) Ag NPs/rGO composites prepared irradiated by 300 mW laser for 1 h with Ag ion concentration of 1 mM, (iv) irradiated by 300 mW laser for 2 h with Ag ion concentration of 1 mM and (v) irradiated by 300 mW laser for 15 min with Ag ion concentration of 5 mM.

Firstly, we synthesized Ag NPs/rGO composites using the LAL method. The effect of laser power, irritation time and Ag ion concentration on the optical property and morphology of the products are systematically studied. Figure 2*a* shows the absorption spectra of GO, rGO and Ag NPs/rGO prepared with different irradiation time. The laser power is fixed at 300** **mW, and the Ag ion concentration is 1** **mM. From the figure, we can see that GO shows a characteristic shoulder at 305** **nm attributed to the *n* → π* transitions of C=O bonds [33]. As a comparison, the absorption shoulder at 305** **nm of rGO disappears and the absorption in the whole visible light range increases after laser irradiation, indicating the reduction in GO (the colour change of GO before and after laser irradiation are given by inset (i) and (ii) of figure 2*d*). When GO solution mixed with Ag ions is reduced (as shown by inset (iii) of figure 2*d*), an obvious absorption peak at 420** **nm is observed which can be attributed to the surface plasmon resonance (SPR) of Ag NPs [13,34]. The SPR peak of Ag NPs is enhanced by increasing the irradiation time, indicating that the reduction degree of the Ag ions is increased. Besides that, the laser power also influences the formation of the Ag NPs. Figure 2*b* shows the absorption spectra of the composites prepared with different laser power. The reduction degree increases with increasing the laser power and is saturated at about 300** **mW, indicating the complete reduction in the Ag ions. It should be noted that too long time or too high laser power irradiation will cause the fragmentization of the GO [35,36].

Figure 2*c* shows the absorption spectra of the composites prepared by adding Ag ions with different concentration. The SPR peak of Ag NPs increased with increasing the Ag ion concentration, and saturated at about 1** **mM. The amount of Ag ions will decide the doping concentration of Ag NPs. However, when Ag ions are more than a certain quantity, a mass of aggregation is observed. The inset (v) of figure 2*d* shows the product of the composites with Ag ions of 5** **mM after 300** **mW laser ablation. An obvious precipitate is found at the bottom of the cuvette, although the irradiation time is set at only 15** **min.

**Figure 3.**
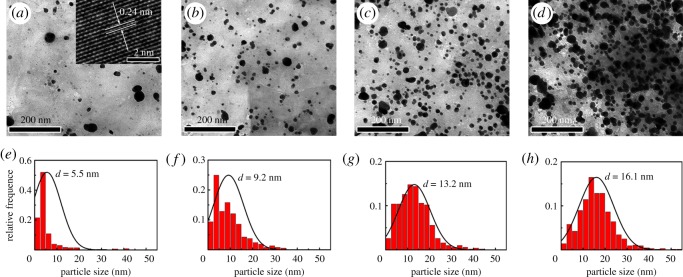
TEM images of the Ag NPs/rGO composites prepared with irradiation times of (*a*) 10 min, (*b*) 20 min, (*c*) 30 min and (*d*) 1 h. (*e*)–(*h*) The size distribution of the Ag NPs evaluated from (*a*) to (*d*). The inset shows the HRTEM image of Ag NPs.

Figure 3*a–d* shows the TEM images of Ag NPs/rGO composites prepared with different irradiating times. The Ag ion concentration is fixed at 1** **mM and the laser power is adjusted to 300** **mW. The inset of figure 3*a* indicates the HRTEM image of Ag NPs. Obvious lattice fringes with the spacing of 0.24** **nm are observed, which can be attributed to the (111) plane of the Ag crystallite [13,34]. Figure 3*e–h* shows the corresponding size distributions of the Ag NPs given in figure 3*a–d*. After 10** **min of irradiation, Ag NPs have been obviously produced. The size of the particles is mainly concentrated at 2–10** **nm, while a few large particles with a size of 20–40** **nm are also observed. With increasing the irradiation time, the ratio of large particles increases and the size distribution of the particles becomes more uniform. These results accord well with the absorption spectra given in figure 2*a*. By prolonging the irradiation time, the amount of the NPs increases, inducing the enhancement of the SPR peak of Ag NPs. After 1 h of irradiation, Ag NPs with high concentration are well dispersed on the rGO nanosheets, and the average size is evaluated to be about 16.1** **nm.

**Figure 4.**
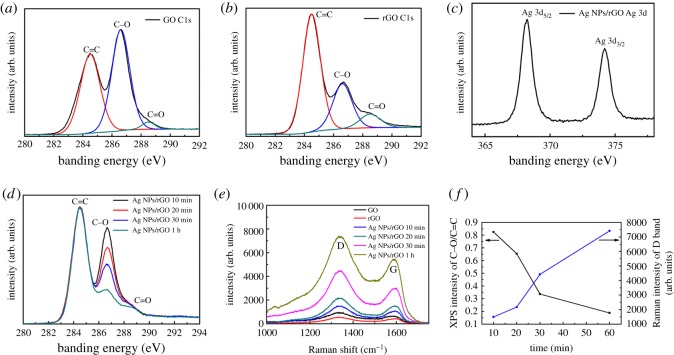
XPS spectra of (*a*) C 1s of GO, (*b*) C 1s of rGO, (*c*) Ag 3d of Ag NPs/rGO, and (*d*) C 1s of Ag NPs/rGO prepared with different irradiation times. (*e*) Raman spectra of GO, rGO and Ag NPs/rGO composites. (*f*) XPS intensity ratio of the C–O to C=C band, and the Raman intensity of the D band of graphene in different composites.

XPS and Raman spectra are explored to study the chemical structure of the Ag NPs/rGO composites. Figure 4*a*,*b* shows the C 1s XPS spectra of GO and rGO, respectively. In both samples, peaks at 284.5, 286.6 and 288.5** **eV assigned to C=C, C–O and C=O groups are observed [33]. The difference lies that, the percentage of C=C and C–O for the GO are calculated to be 44.87 and 52.51%, while those for the rGO are 64.57 and 26.81%. Figure 4*c* displays the Ag 3d XPS spectra for Ag NPs/rGO. The two peaks centred at 368.2 and 374.2** **eV correspond to the banding energies of Ag 3d_5/2_ and Ag 3d_3/2_ of Ag, respectively. Figure 4*d* shows the evolution of the C 1s XPS spectra of the composite prepared with different irradiation times. It is evident that C–O groups decrease with prolonging the irradiation time. Figure 4*e* gives the Raman spectra of GO, rGO and Ag NPs/rGO samples. Two characteristic peaks corresponding to the D and G bands are observed. The D band peak near 1330 cm^−1^ is attributed to disorder or defects in carbon atoms, while the G band peak near 1590 cm^−1^ is attributed to the sp^2^ in-plane vibration of carbon atoms [22]. Owing to the surface enhancement effect of the Ag NPs, Raman spectrum of Ag NPs/rGO is significantly enhanced compared to GO and rGO [37]. Figure 4*f* summarizes the XPS intensity ratio of the C–O to C=C band, and the Raman intensity of the D band of graphene in different composites. With increasing the irradiation time, the intensity ratio of C–O to C=C decreases gradually, indicating that the reduction degree of GO is enhanced. Besides that, as more Ag NPs are produced by prolonging the irradiation time, the surface enhancement effect of the Ag NPs is strengthened, and as a result, the D band (as well as the G band) Raman signal of graphene increases.

### 3.2. Optical limiting properties and mechanisms of the graphene samples

**Figure 5.**
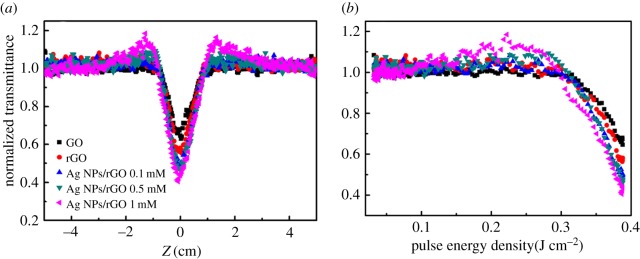
(*a*) Z-scan results of GO, rGO and Ag NPs/rGO solutions. (*b*) Nonlinear transmittance of the samples as functions of the input laser energy density.

The nonlinear OL property of the as-prepared Ag NPs/rGO hybrids is studied using the Z-scan technique. To control the doping concentration of the Ag NPs, the concentrations of added Ag ions vary from 0.1 to 1** **mM, while the laser power and irradiation time is fixed at 300** **mW and 1 h, respectively. In the experiments, a nanosecond laser at 532** **nm was focused with a lens of 200** **mm focal length with a pulse energy of 100 µJ. The linear transmittance of the samples are all adjusted to 80%. Figure 5*a* shows the normalized nonlinear transmittances as functions of the *z*-position in GO, rGO and Ag NPs/rGO composites. It is clearly seen that the transmittances in all samples decreased with increasing the incident light intensity, exhibiting significant OL properties. Compared with GO, the OL property of rGO is enhanced, while those for Ag NPs/rGO is further improved with increasing the Ag NPs concentration. Figure 5*b* shows the nonlinear transmittances as functions of the incident laser energy density extracted from figure 5*a*. The OL threshold of Ag NPs/rGO with 1** **mM Ag ions is estimated to be about 0.38 J cm^−2^, which is much lower than the graphene dispersions given in some previous reports [11].

**Figure 6.**
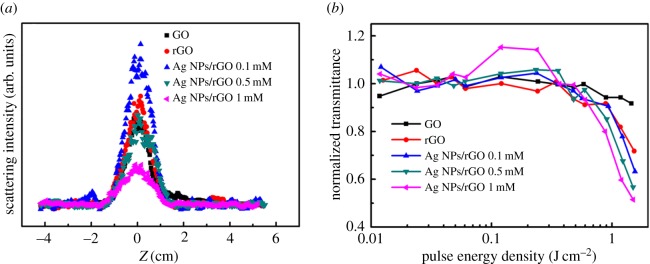
(*a*) NLS and (*b*) NLA measurement results in GO, rGO and Ag NPs/rGO solutions.

In the OL process of graphene materials, two main nonlinear effects, i.e. NLS and NLA, might participate. In order to determine the OL mechanism of these solutions, the NLS light intensity as a function of the incident light intensity is measured with the incident pulse energy of 100 µJ. The results in figure 6*a* indicate the onset of the increase in scattered signals is synchronous with the onset of the decrease in transmission for all the solutions. This demonstrates that NLS effect plays an important role in the OL process of the materials. We can clearly see that the NLS intensity is enhanced after GO is reduced to rGO. For Ag NPs/rGO composites, the NLS intensity is increased compared with rGO when the concentration of Ag ions is 0.1 mM. As the used laser at 532 nm is at the band edge of the SPR peak of Ag NPs/rGO, more laser energy can be absorbed and converted into heat energy. Because the NLS effect mainly originates from thermally induced microbubbles and/or microplasmas in solvent [11], more microbubbles and/or microplasmas can be induced in Ag NPs/rGO which results in an enhanced NLS intensity at a low concentration of Ag NPs. However, the NLS intensity of Ag NPs/rGO is decreased when Ag ion concentration is increased to 0.5 and 1 mM. We speculate that the NLA effect might be enhanced when the concentration of Ag NPs increases, and as a result, the energy converted to heat reduces, causing the decrease in NLS intensity.

To confirm the contribution of the NLA effect to the OL behaviour of the materials, NLA effects in the samples are measured by collecting the scattering light using a lens. Using this arrangement, the collected scattering light and the transmitted light will incident into the detector together. Therefore, the contribution of the NLS effect to the OL behaviour can be ruled out, and the decrease in the transmittance is mainly attributed to the NLA effect. Figure 6*b* shows the nonlinear transmittance as a function of the incident light intensity induced by the NLA effect. It is clearly seen that both rGO and Ag NPs/rGO exhibit obvious OL behaviour, while the transmittances change little in GO. Besides, the NLA effect is gradually increased when the concentration of Ag ions is increased from 0.1 to 1 mM. When focused laser irradiates into the nanocomposites, Ag NPs absorbs laser light and free electrons are generated. The photogenerated electrons could efficiently transfer from Ag NPs to rGO, suppressing the charge recombination and producing a charge-separated excited state. Therefore, it is believed that the photo-induced charge transfer from Ag NPs to rGO in the Ag NPs/rGO composites lead to the enhanced NLA [21]. Finally, we can conclude that both NLA and NLS effects contribute to the OL effect in rGO and Ag NPs/rGO composites, while only NLS contributes to those in GO. With increasing the Ag NPs concentration, the NLA-induced OL property can be effectively enhanced. As the NLS effect mainly originates from thermally induced microbubbles and/or microplasmas in solvent, the enhanced NLA effect in Ag NPs/rGO makes it possible to manufacture solid-state optical limiter, improving the practicality of graphene in the field of OL.

## 4. Conclusion

We successfully synthesized Ag NPs/rGO nanocomposites by ablating the mixed aqueous solutions of AgNO_3_ and GO using femtosecond laser pulses. The sizes of Ag NPs we obtained were mainly concentrated in 2–40** **nm. The significant enhancement of NLO response of nanocomposites was observed by the Z-scan method using a nanosecond laser at 532** **nm. The results showed that Ag NPs/rGO solution exhibited strong OL properties. The NLS and NLA measurements indicated that the main OL mechanism of GO was NLS, while the rGO and Ag NPs/rGO exhibited both strong NLS and NLA. The enhanced NLA effect in the hybrid makes it possible to manufacture solid-state optical limiter, improving the practicality of the material in the OL area.

## Data accessibility

Our data are deposited at Dryad: https://doi.org/10.5061/dryad.6j6v707 [38].
